# Crude Fucoidan Extracts Impair Angiogenesis in Models Relevant for Bone Regeneration and Osteosarcoma via Reduction of VEGF and SDF-1

**DOI:** 10.3390/md15060186

**Published:** 2017-06-20

**Authors:** Fanlu Wang, Harald Schmidt, Dijana Pavleska, Thees Wermann, Andreas Seekamp, Sabine Fuchs

**Affiliations:** 1 Experimental Trauma Surgery, University Medical Center Schleswig-Holstein, 24105 Kiel, Germany; Fanlu.Wang@uksh.de (F.W.); Dijana.Pavleska@uksh.de (D.P.); thees-marten-wermann@web.de (T.W.); Andreas.Seekamp@uksh.de (A.S.); 2 MetaPhysiol, Am Römerberg, 55270 Essenheim, Germany; schmidt@metaphysiol.de

**Keywords:** fucoidan, mesenchymal stem cells, outgrowth endothelial cells, co-culture, angiogenesis, bone formation, osteosarcoma, VEGF, SDF-1

## Abstract

The marine origin polysaccharide fucoidan combines multiple biological activities. As demonstrated by various studies in vitro and in vivo, fucoidans show anti-viral, anti-tumor, anti-oxidant, anti-inflammatory and anti-coagulant properties, although the detailed molecular action remains to be elucidated. The aim of the present study is to assess the impact of crude fucoidan extracts, on the formation of vascular structures in co-culture models relevant for bone vascularization during bone repair and for vascularization processes in osteosarcoma. The co-cultures consisted of bone marrow derived mesenchymal stem cells, respectively the osteosarcoma cell line MG63, and human blood derived outgrowth endothelial cells (OEC). The concentration dependent effects on the metabolic activity on endothelial cells and osteoblast cells were first assessed using monocultures of OEC, MSC and MG63 suggesting a concentration of 100 µg/mL as a suitable concentration for further experiments. In co-cultures fucoidan significantly reduced angiogenesis in MSC/OEC but also in MG63/OEC co-cultures suggesting a potential application of fucoidan to lower the vascularization in bone tumors such as osteosarcoma. This was associated with a decrease in VEGF (vascular endothelial growth factor) and SDF-1 (stromal derived factor-1) on the protein level, both related to the control of angiogenesis and furthermore discussed as crucial factors in osteosarcoma progression and metastasis. In terms of bone formation, fucoidan slightly lowered on the calcification process in MSC monocultures and MSC/OEC co-cultures. In summary, these data suggest the suitability of lower fucoidan doses to limit angiogenesis for instance in osteosarcoma.

## 1. Introduction

Vascularization, the formation of blood vessels, plays an essential role in bone repair [1,2,3] and normal bone physiology as well as in bone tumor development [4]. New blood vessel formation from existing vasculature, defined as angiogenesis, is supposed to be the dominant process for neovascularization in mature bone [2]. Nevertheless, a series of studies also imply the contribution of stem cells and progenitor cells such as circulating endothelial progenitor cells in the formation of new blood vessels [5,6]. In bone tumors such as osteosarcomas angiogenesis plays a key role mediating tumor growth and formation of metastasis [7]. In principle, molecular mechanisms guiding tumor-induced angiogenesis and neovascularization during bone regeneration seem to be at least similar [8]. In this context, co-culture systems have been shown to serve as highly relevant in vitro models to study the physiological and pathological processes of angiogenesis in the bone, permitting the study of reciprocal reactions between bone cells [9,10], endothelial cells and circulating cells at cellular and molecular levels [11,12]. In addition, these models also serve as experimental platforms to test drug delivery options [13] or compounds modulating the vascularization process with the aim to detect specific effects on single cell types and effector molecules.

The aim of this study is to assess the impact of commercially available crude fucoidan extracts, a mixture of sulfated polysaccharide from seaweed, on the formation of vascular structures in co-culture models relevant for bone vascularization during bone repair and vascularization processes in osteosarcoma. The co-cultures consisted of bone marrow derived mesenchymal stem cells, respectively the osteosarcoma cell line MG63, and human blood derived endothelial cells (Outgrowth endothelial cells (OEC)) [14,15]. As demonstrated in a large number of in vitro and in vivo, studies fucoidans shows anti-viral [16,17], anti-tumor [18,19,20], anti-oxidant [21], anti-inflammatory [22] and anti-coagulant [23] properties.

Nevertheless, the biological effects of fucoidan are closely related to its chemical structure and composition. The effects depend on the molecular weight, sulfation degree, molecular geometry and sugar composition [24,25]. In addition, fucoidan is often a crude mixture of sulfated polysaccharides and this chemical heterogeneity of fucoidans is largely depending on the source, the extracting method and even the time point of harvesting. This results in difficulties to correlate specific chemical features with the associated bioactivities [25,26]. Commercial fucoidans from fucus vesiculosus are known as a crude extract and have been used in biological studies [27]. This extract includes a wide spectrum fucoidans with different molecular weights and chemical structures [28]. Recently it has been shown that this type of fucoidan extract provides anti-angiogenic properties in a macular degeneration model by suppressing the VEGF expression and secretion of retinal pigment epithelial cell lines and thus limits the angiogenic activity of blood derived outgrowth endothelial cells (OECs) [29]. Accordingly, this result also implies a potential value of fucoidan to suppress angiogenesis in the bone or in bone tumors, respectively.

VEGF, is a key regulatory molecule in angiogenesis promoting adhesion, migration, proliferation and survival of endothelial cells [30] with a high impact specifically in the bone [31]. For these reasons, VEGF has been studied as a common target to control angiogenesis in general and specifically to control tumor growth [32,33]. In addition, fucoidan has been shown to modulate the CXCR4/SDF-1 pathway [34] guiding the recruitment of different cell types in the bone [35] and in many other tissues. This CXCR4/SDF-1 pathway is relevant for attraction of stem cells [36] as well for many other cells relevant in tumor progression [37,38,39,40] and control of angiogenesis in general [41,42,43].

In this study, we applied fucoidan from fucus vesiculosus to co-cultures of human peripheral blood derived outgrowth endothelial cells (OECs) and human bone marrow derived mesenchymal stem cells (MSCs) or respectively human bone sarcoma cell line (MG63). We investigated the impact of fucoidan supplemented medium on the formation of vascular structures and correlated these findings with the expression and secretion of VEGF and other relevant molecules such as Angiopoietins (angiopoietine-1 and angiopoietin 2) involved in the control of the angiogenesis [44]. Furthermore, we investigated the consequences of fucoidan treatment on molecules involved in bone formation and the recruitment of circulating cells via the CXCR4/SDF-1 pathway. In addition, we assessed the impact of fucoidan on molecules involved in the bone function and the calcification process.

## 2. Materials and Methods

### 2.1. Ethical Approval for the Use of Human Cells

The use of MSC and OEC isolated from human tissues was approved by the local ethical advisory boards including the consent from the individual donors.

### 2.2. Isolation and Culture of MSCs

Human mesenchymal stem cells (MSCs) loosely attached to cancellous bone structures were collected from bone fragments by washing in phosphate buffered saline (PBS) (Gibco, Darmstadt, Germany) as described before [16]. Following washing the cells in PBS were collected. After centrifugation, cells were resuspended in Dulbecco’s Medium Essential Medium (DMEM)/Ham F-12 (Biochrom, Berlin, Germany) supplemented with 20% fetal bovine serum (FBS) (Sigma, Taufkirchen, Germany) and 1% penicillin/streptomycin (Pen/Strep) (Biochrom, Berlin, Germany) and seeded at a density of 2 × 10^6^ cells/cm^2^ in collagen type I (Corning, Bedford, MA, USA) coated flasks. MSCs were expanded by subculturing and further cultivated in osteogenic differentiation medium (ODM) consisting of DMEM/Ham F-12; 0.1 μM dexamethasone (Sigma-Aldrich, St. Louis, MO, USA); 10 mM β-glycerol phosphate (Sigma-Aldrich); 50 μM ascorbate-2-phosphate (Sigma-Aldrich); 10% FBS, and 1% Pen/Strep for 2 weeks to obtain osteogenic lineage cells. Cells were derived from several MSC donors at the age of 69 (m), 53 (f), 70 (f) and 73 (f).

### 2.3. Isolation and Expansion of OECs

Human outgrowth endothelial cells (OECs) are derived from human peripheral blood, and have been isolated and cultured by the methods described previously [15,45]. In brief, human mononuclear cells were isolated from buffy coat by gradient centrifugation with Biocoll (Biochrom, Berlin, Germany). Cells were resuspended in endothelial cell growth medium-2 (EGM-2) (Lonza Walkersville, MD, USA) with supplements from the kit, 5% FBS and 1% Pen/Strep. Cells were seeded in collagen type I coated 24-well-plates at a density of 2.6 × 10^6^ cells/cm^2^. After 7 days, cells were subcultured to new collagen type I coated 24-well-plates at a density of 2.6 × 10^5^ cells/cm^2^. Cobblestone-like OECs were observed within 2–3 weeks. OECs were subcultured and expanded to be used in the experiments.

### 2.4. Fucoidan Treatment of Mono and Co-Cultures

MG63 osteosarcoma cells were cultivated in Dulbecco’s MEM (Biochrom, Berlin, Germany) supplemented with 10% FBS, 1% l-Glutamine (Gibco, Darmstadt, Germany) and 1% Pen/Strep. Cell subculture was performed after reaching 80–90% confluence. MG63 cells with different passage numbers within the range from 15 to 23 were used in mono-cultures and co-cultures with OECs.

For mono-cultures of MSC, MG63 or OEC, cells were seeded in fibronectin (Millipore, Temecula, CA, USA) coated 24 well-plates at a density of 40.000 cells/cm^2^. After one day, growth medium with different concentrations fucoidan from fucus vesiculosus (Sigma-Aldrich, Taufkirchen, Germany) was applied to the growth medium or cells were maintained in normal growth medium as controls.

For co-cultures the seeding of MSC and MG63 was performed as described above followed by adding OEC on the next day at a density of 40.000 cells/cm^2^. EGM-2 medium supplemented with 100 μg/mL fucoidan was applied to co-cultures one day after seeding of OECs, or cells were grown in EGM-2 as controls. Medium changes were performed every 3 days for both mono- and co-cultures.

### 2.5. MTS Cell Metabolic Activity Assay

The metabolic activity of MSC and OEC mono-cultures in response to fucoidan treatment was determined with CellTiter 96^®^ AQueous One Solution Cell Proliferation Assay (Promega, Madison, WI, USA) as described by the instruction from the supplier. MSC, MG63 or OECs respectively were seeded on 96-well-plates at a density of 40.000 cells/cm^2^. Fucoidan treatment was performed as described above. The medium was replaced with MTS solution and incubated at 37 °C for 2 h according to the manufacturer’s instruction. The absorbance at 490 nm was measured in a microplate reader (Apollo, Berthold Technologies, Bad Wildbad, Germany). The cell metabolic activities in percentage were calculated as the mean value normalized to control group (100%) after subtracting the absorbance of the blanks.

### 2.6. Immunofluorescence Staining and Visualization of Angiogenic Structures

Co-cultures grown on Thermanox coverslips were immunostained for the endothelial marker VE-Cadherin and the angiogenesis and cell recruitment related marker CXCR4. The MSC/OEC and MG63/OEC co-cultures were fixed with 4% paraformaldehyde solution in PBS (Affymetrix, Cleveland, OH, USA) on day 10 (and day 7, Supplemental Data). Cells were washed 3 times for 5 min PBS and then permeablized with 0.5% Triton^®^ X-100 (Sigma-Aldrich, Taufkirchen, Germany). Then cells were incubated with primary antibodies VE-Cadherin (R&D, Minneapolis, MN, USA) and CXCR4 (R&D, derived in mouse) both 1:50 diluted in PBS with 1% bovine serum albumin (BSA) (Millipore, Kankakee, IL, USA) for 1.5 h. After washing with PBS, cells were incubated with the secondary antibodies Alexa 555 donkey anti goat (Invitrogen, Eugene, OR, USA) and Alexa 488 rabbit anti-mouse (Invitrogen) 1:1000 diluted in 1% BSA for 30 min followed by 5 min incubation with Hoechst diluted in PBS. Samples were mounted with Fluoromount Aqueous Mounting Medium (Sigma-Aldrich, St. Louis, MO, USA) before visualization with confocal laser scanning microscopy (CLSM, LSM 510 Meta, Zeiss, Oberkochen, Germany).

### 2.7. Quantification of Agiogenesis

The immunofluorescense-stained cells (co-cultured MSC/OEC and MG63/OEC) were photographed (20-fold magnification) and images were analyzed using ImageJ 1.43 as described previously [46]. In brief, the images were segmented semi automatically and area and length of tube-like structures were quantified for samples with and without (control) addition of Fucoidan on day 10.

### 2.8. Quantification of DNA Content

The potential influence of fucoidan on cell growth was evaluated by DNA assessment. Cells grown in the wells were trypsinized, the pellet was harvested by centrifugation at 2000 g for 5 min and finally resuspended in a microtube with 1 mL deionized water. Cell membrane was destroyed by freeze-thaw cycles and sonication. The dsDNA content released in the aqueous solution was examined with Quant-iT PicoGreen dsDNA assay kit (Molecular probes, Eugene, OR, USA). Each sample or standard was prepared in triplicate. DNA amount was determined by fluorescence using a microplate reader (TECAN, Maennedorf, Switzerland) at 485/535 nm of excitation/emission wavelength according to a standard curve.

### 2.9. Gene Expression Analysis

Cells were lysed with cell lysis buffer from peqGOLD Total RNA Kit (VWR peqlab, Erlangen, Germany). Total RNA was isolated according to the manufacturer’s protocol. The RNA concentration was measured with a NanoDrop (Thermo Fisher, Erlangen, Germany). Then 1 μg of total RNA from each sample was transcribed to cDNA with high capacity RNA-to cDNA Kit (Applied Biosystems, Carlsbad, CA, USA). PCR was performed for primers as indicated in Table 1 using RPL13a as internal control. Quantitative real-time PCR was carried out using a total volume of 25 μL for each reaction and SYBR^®^ Select Master Mix (Applied biosystems, Austin, TX, USA), cDNA QuantiTect^®^ Primer Assay (Qiagen, Hilden, Germany) or CD31(Eurofins, www.eurofins.com), RNase free water (Qiagen), and cDNA were added. The mixtures were preheated to 50 °C for 20 min and 95 °C for 20 min followed by 40 cycles of step 1: 95° for 15 s and step 2: 60° for 60 s. The relative gene expression was calculated with ΔΔcT method. Fucoidan treated groups were compared to untreated conditions which were normalized to 1 as control.

### 2.10. Enzyme Linked Immunosorbent Assay (ELISA)

The supernatants of mono-/co-cultures were collected at day 7 (not shown) or 10 to perform ELISA for VEGF, Angiopoietin-1 and Angiopoietin-2 with DuoSet ELISA Development kit (R&D, Minneapolis, MN, USA) according to manufacturer’s protocols. Absorbance was detected using a microplate reader (Apollo) at 450 nm with a reference wavelength of 560 nm. Samples from at least 3 donor sets were analyzed in triplicate. The amount of proteins from cellular supernatant was normalized to DNA content. The ELISA results are presented in relative values of M_protein/DNA_ in percentage compared to the control group calculated with the formula [M_sample_/M_control_] × 100%.

### 2.11. Quantification of Mineralization by Alizarin Red Staining and Quantification

To quantitatively determine the impact of fucoidan on the calcification in the co-cultures, 1 mL of 40 mM Alizarin Red S Stain Solution (Millipore, Billerica, MA, USA) was applied to 4% PFA fixed cells at day 14 and incubated for 30 min. Cells were washed with distilled water until the wash solution was clear then incubated with 10% cetylpyridinium chloride (Roth, Karlsruhe, Germany) overnight to extract the Alizarin Red dyes combined with mineralized ECM. Alizarin Red supernatants collected from stained cell layers and standards were added in a 96-well-plate and read at a wavelength of 560 nm with a microplate reader (Apollo) for quantification.

### 2.12. Statistical Analysis

All experiments mentioned above were carried out with at least 3 different donor sets. The statistical significance between fucoidan treated and control group was assessed as shown for the individual graphs with student’s *t*-test or ANOVA using Graphpad Prism 5. *p* < 0.05 (* *p* < 0.05, ** *p* < 0.01, *** *p* < 0.001) was considered as statistically significant difference.

## 3. Results

### 3.1. The Metabolic Activity of Individual Cell Types in Response to Fucoidan Dose

**Figure 1:**
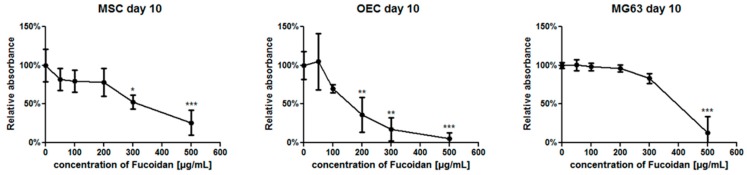
Effect of different fucoidan concentrations on the metabolic activity of OEC, MSC and MG63. Data are depicted in percent in relation to untreated groups used as controls (100%), 1-way ANOVA. * *p* < 0.05, ** *p* < 0.01, *** *p* < 0.001 was considered as statistically significant difference.

The MTS assays were performed to examine a potential effect of fucoidan on the metabolic activities of MSC, MG63 and OEC in monocultures at day 10 (Figure 1) using different concentrations of fucoidan. MTS absorbance values were depicted as relative changes of fucoidan treated groups compared to untreated controls (100%).

For 100 µg/mL (Figure 1), the metabolic activity of MSC and OECs showed only a slight but no significant reduction in fucoidan treated group compared to controls. The metabolic activity was further reduced in groups treated with higher concentrations of fucoidan. In accordance with first effects of fucoidan on OECs at a fucoidan concentration of 200 µg/mL, OECs seemed to be more sensitive compared to MSC (significant effects observed at 300 µg/mL) whereas MG63 seemed to tolerate higher concentrations of fucoidan (significant effects at 500 µg/mL). In accordance with these observations, all further experiments to assess angiogenesis as well as osteogenesis were performed with a fucoidan concentration of 100 µg/mL.

### 3.2. Angiogenic Structures of OECs in Co-Cultures

**Figure 2:**
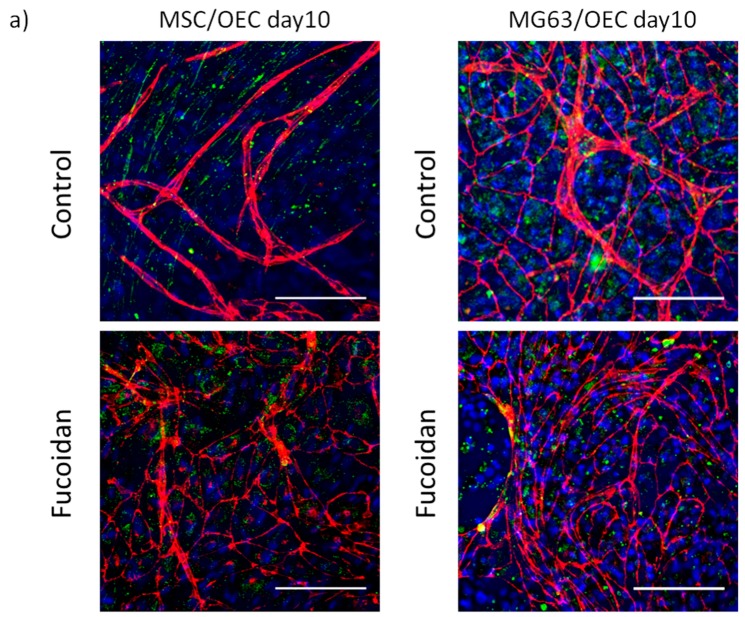

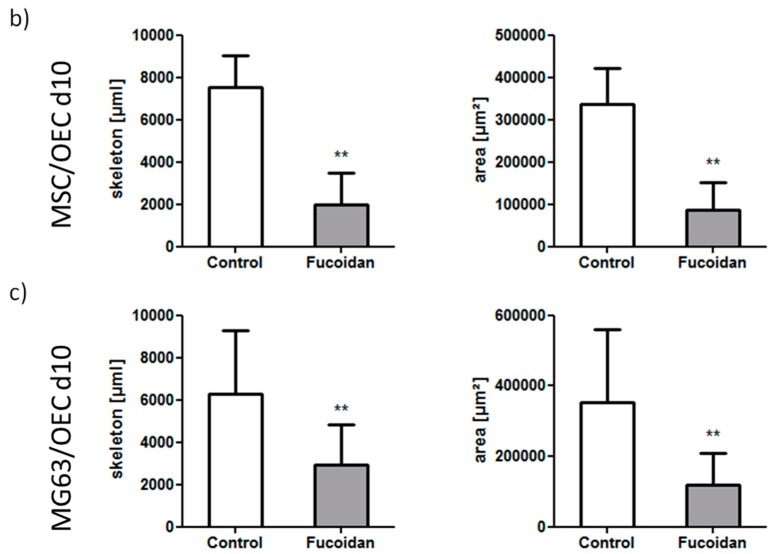
Effect of fucoidan on the morphology and pro-angiogenic structures in co-cultures. (**a**) Confocal laser scanning microscopy of MSC/OEC and MG63/OEC co-cultures on day 10. VE-Cadherin is depicted in red, green channel represents staining for CXCR4 and nuclei are depicted in blue. The scale bar represents 150 μm. (**b**,**c**) Quantitative analysis of angiogenic structures depicting the skeleton length and the area of angiogenic structures for MSC/OEC co-cultures (**b**) and MG63/OEC co-cultures (**c**). The results are given as means ± S.D. and significant differences were calculated with Graph Pad Prism using an unpaired *t*-test (*p* < 0.05 * and *p* < 0.01 **) for equal variances as verified with a variance ratio analysis (*F*-test, *p* > 0.05). For unequal variances (*F*-test, *p* < 0.05) data were analyzed using the unpaired *t*-test with Welch’s correction.

The morphology of OECs and the formation of angiogenic structures by OECs in co-cultures were visualized with confocal microscopy after immunostaining with endothelial marker VE-Cadherin (Figure 2a depicted in red) at day 10 (day 7 see Supplemental Data Figure S1). In addition, the samples were stained for stromal-derived factor receptor CXCR4 (Figure 2a, depicted in green, nuclear counterstain, blue). For MSC/OEC co-cultures, OECs in the untreated control group showed elongated cell shape and were aligned into tubular structures typical for pro-angiogenic structures indicated in Figure 2a. In contrast, fewer pro-angiogenic structures were observed after fucoidan treatment (100 µg/mL) and OECs remained mainly organized as monolayers with distinct cell-cell contacts as indicated by VE-Cadherin staining, although the formation of angiogenic structures was not completely blocked after fucoidan treatment.

Similar to MSC/OEC co-cultures, OECs in co-cultures with MG63 were characterized by the formation of complex angiogenic structures at day 10 in untreated control groups as shown in Figure 2a. In fucoidan treated groups, only a few elongated angiogenic structures of OECs were observed mainly at the borders of OEC cell patches.

To quantify the effects of fucoidan on the formation of angiogenic structures, we performed quantitative image analysis as depicted in Figure 2b,c. For both types of co-cultures the addition of fucoidan to the culture medium resulted in a significant decrease in length and the area of angiogenic structures thus showing anti-angiogenic properties of fucoidan in co-culture models relevant for normal bone physiology or osteosarcoma, respectively.

### 3.3. DNA Quantification

**Figure 3:**
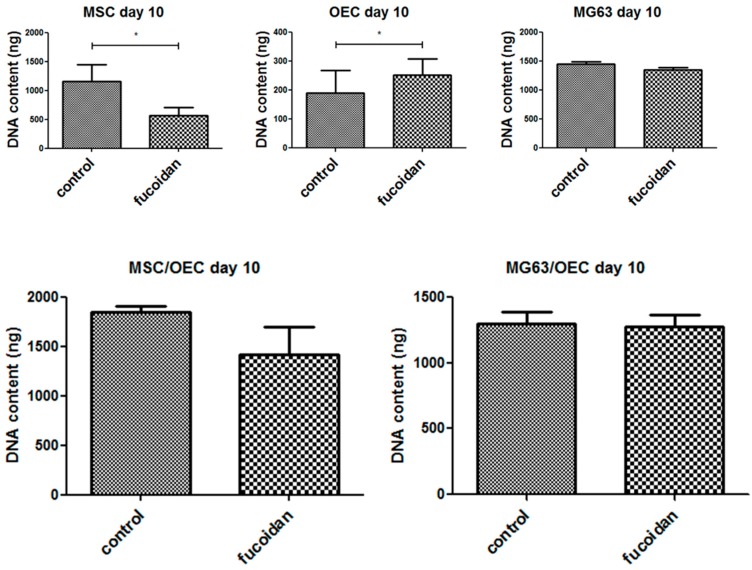
Effect of fucoidan on cell proliferation depicting the DNA content of MSC, OEC and MG63 mono-cultures (paired *t*-test) as well as MSC/OEC and MG63/OEC co-cultures; * *p* < 0.05, *n* = 3, (unpaired *t*-test).

In order to gain a deeper insight in the action of fucoidan and potential effects on cell growth, the total DNA content, as indicator for cellular proliferation, was analyzed in mono-cultures of OEC, MSC and MG63 and both groups of co-cultures using a picogreen based assay (Figure 3). The Total DNA content analyzed at day 10 decreased significantly for MSC monocultures but increased for mono-cultures of OEC in response to fucoidan treatment, whereas no significant difference was observed for MG63. For both co-cultures the DNA content slightly decreased in fucoidan treated groups but this trend was not statistically significant in the co-cultures.

### 3.4. Quantitative Assessment of Gene Expression of Mono-/Co-Cultures

**Figure 4:**
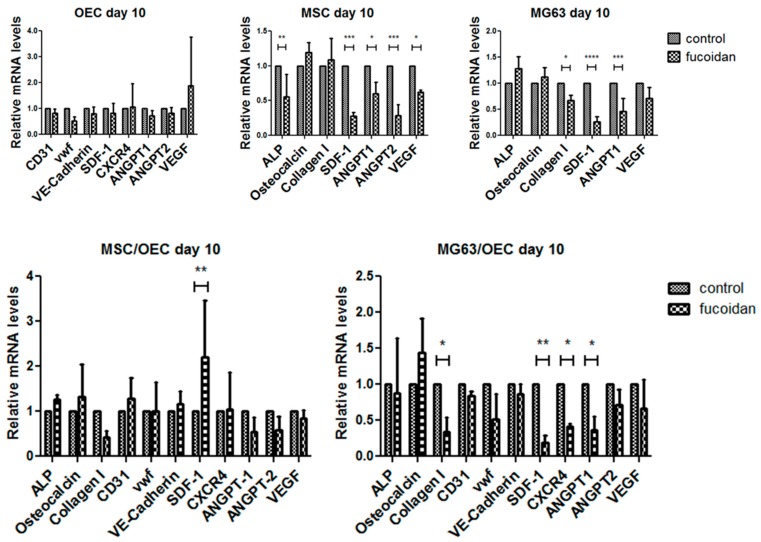
Relative gene expression for osteogenic markers (ALP, osteocalcin, collagen I), growth factors (SDF-1, CXCR4, ANGPT1, ANGPT2, VEGF) and endothelial markers (CD31, vWf, VE-Cadherin) evaluated by semi-quantitative RT-PCR for mono-cultures of OEC, MSC and MG63 as well as MSC/OEC and MG63/OEC and co-cultures on day 10. * *p* < 0.05, ** *p* < 0.01, *** *p* < 0.001, **** *p* < 0.0001, *n* = 3, 2-way ANOVA.

As a next step we performed semi-quantitative real time PCR studies for mono- and co-cultures (Figure 4) in control and fucoidan treated groups and analyzed the relative gene expression of molecules associated with bone formation and osteogenic differentiation, factors involved in control of angiogenesis and stem cell recruitment, as well as endothelial markers.

In OECs no significant effects in the investigated genes in response to fucoidan treatment could be observed. Nevertheless, in the osteogenic cells a significant impact of fucoidan treatment was observed. This includes a significant downregulation of the molecules Ang-1, and VEGF, involved in the modulation of angiogenesis by MSCs [15,47,48] via paracrine factors. Similar effects were also observed for the osteosarcoma cell line MG63 although the effect on VEGF was not significant for MG63. In addition, the fucoidan treatment resulted in a significant downregulation of SDF-1 in both MSC and MG63 as factors involved in the recruitment of a series of cell types [36,49,50]. Angiopoietin 2 (Ang-2) a proangiogenic factor was tentatively downregulated in the co-cultures. In terms of effects of the fucoidan treatment on osteogenic differentiation markers, the results differed in the MSC and the osteosarcoma cell line MG63 showing significant downregulation for alkaline phosphatase only in MSC and significant downregulation of collagen type 1 only in MG63. In co-cultures of MG63 and OEC again SDF-1 and its corresponding receptor CXCR4 levels were reduced in response to fucoidan, as well collagen type 1 and angiopoietin-1.

### 3.5. Analysis of Angiogenesis Relevant Factors in Culture Supernatants by Enzyme-Linked Immunosorbent Assay (ELISA)

In accordance with the results from the PCR the quantity of angiogenesis relevant factors including VEGF, ANGPT-1, ANGPT-2 and SDF-1 involved in cell recruitment and osteosarcoma progression in supernatant was measured by ELISA to gain insight on the effects of fucoidan on the protein level.

**Figure 5:**
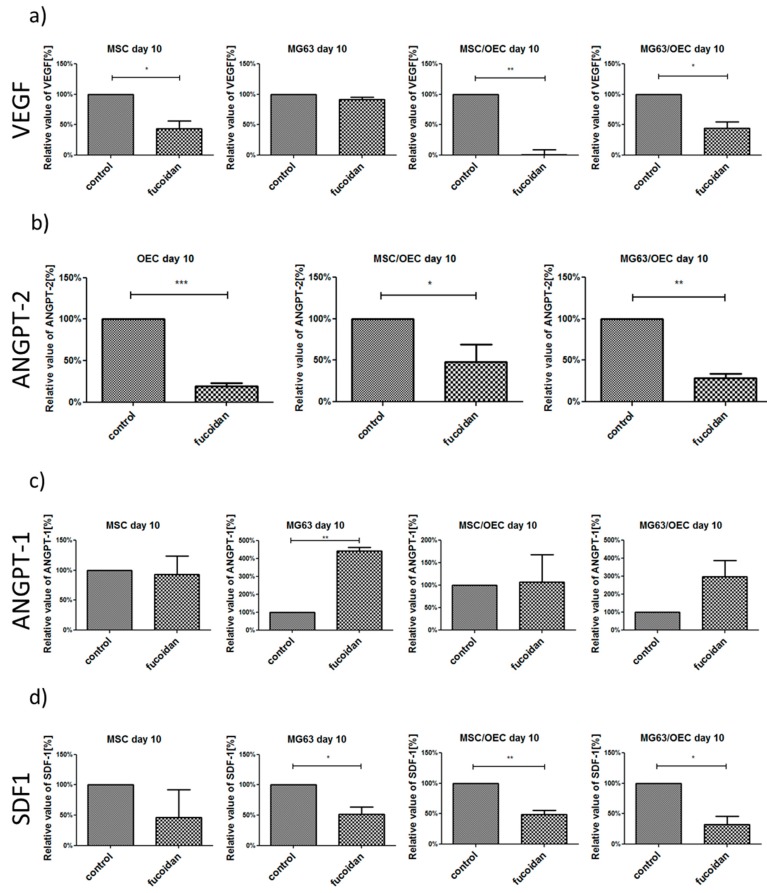
Enzyme-linked immunosorbent assay. For (**a**) VEGF, (**b**) ANGPT-2, (**c**) ANGPT-1 and (**d**) SDF-1 in MSC, MG63 mono- and co-cultures on day 10. The ELISA data are presented in relative values of M_protein/DNA_ in percentage compared to the control group calculated with the formula [M_sample_/M_control_] × 100%. * *p* < 0.05, ** *p* < 0.01, *** *p* < 0.001. *n* = 3; paired *t*-test.

The protein levels in the supernatants were normalized to DNA content and are depicted in % values in relation to the controls (untreated 100%) in Figure 5.

Fucoidan treatment resulted in significantly lower VEGF levels in co-cultures of MSC/OEC and MG63/OEC with similar effects observed for MSC monocultures (Figure 5a) and tentatively for MG63 monocultures. In addition Angiopoietin-2 a proangiogenic factor which is mainly produced by endothelial cells themselves [51] was reduced in response to fucoidan treatment in all samples (MSC and MG63 levels not detectable, data not shown). In contrast, Angiopoietin 1, which binds like the Ang-2 to the Tie-2 receptor and limits the formation of new vascular structures but leads to vascular stabilization, was increased. Finally, SDF-1 mainly associated with the recruitment of stem cells and immune cells in the bone was decreased significantly in response to fucoidan in MG63 monocultures as well as in both types of co-cultures. These reduced protein levels for VEGF, Ang-2 and SDF-1 in co-cultures in response to fucoidan are the determining factors in the physiological process of angiogenesis and cell recruitment in the co-cultures. These processes are mediated by proteins and corresponding receptor mechanisms guiding the cellular response. Potentially conflicting results on the PCR level are most probably due to compensatory upregulation of genes when the protein levels are low.

### 3.6. Quantitative Analysis of Osteogenesis of MSC/OEC Co-Cultures

**Figure 6:**
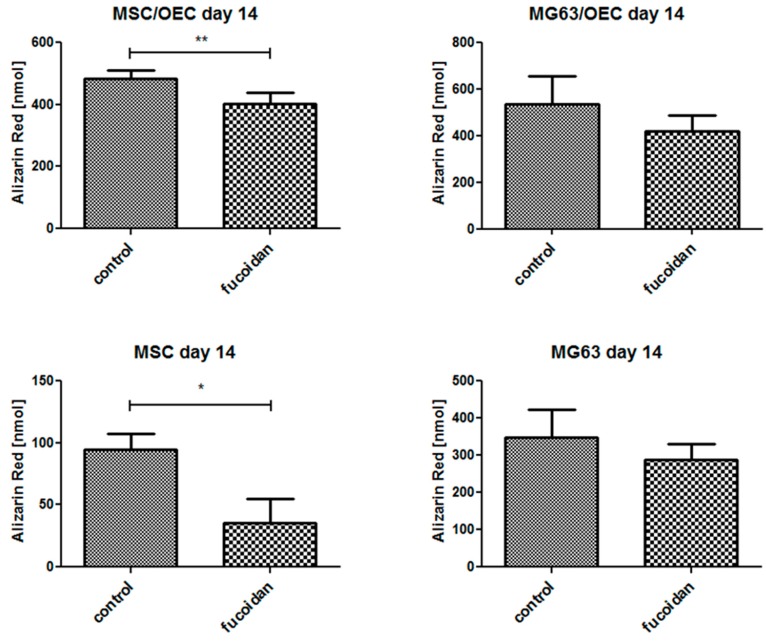
Quantification of Calcification based on Alizarin Red in response to fucoidan for MSC, MG63 mono-cultures and co-cultures at day 14. * *p* < 0.05, ** *p* < 0.01. *n* = 3, paired *t*-test.

In order to assess potential effect of fucoidan on the process of bone formation we assessed the calcification levels as depicted in Figure 6 on day 14 of the cultures. The MSC mono-cultures treated with fucoidan showed a significant reduction of calcification compared to the untreated groups. Similarly, in MSC/OEC co-cultures the calcification of the fucoidan treated group was significantly lower than in the control. For MG63/OEC co-cultures and MG63 monocultures, a similar trend but no significant impact was observed which might be due to the tumor cell characteristics of MG63.

## 4. Discussion

In accordance with the literature fucoidan, a polysaccharide from the brown seaweed fucus vesiculosus, is discussed to modulate the angiogenic process being either applied to enhance angiogenesis for applications in tissue engineering or to restrict the angiogenic process for instance in bone tumors such as osteosarcoma. This pro- or anti-angiogenic effect of fucoidan is dependent on a variety of factors, such as the structural composition. In addition, the effect of fucoidan depends on the kind of application. In this study, we assessed the impact of free fucoidan on the cellular and molecular processes in angiogenesis and osteogenesis taking into account the effects of fucoidan on the individual key cell types and their interaction in co-cultures mediated by growth factors and chemokines.

A recent approach based on fucoidan nanoparticles as a mean to treat osteosarcoma resulted in fucoidan induced apoptosis in primary tumors in vivo and a reduction in metastasis [51]. In addition, cytotoxic effects of fucoidan nanoparticles in osteosarcoma cell lines were detected in this study. In our study, MTS assays have shown that MG63 tolerated higher concentrations of fucoidan compared to the endothelial cells in the systems. Fucoidan concentrations of 100 µg/mL caused no cytotoxic effect. Nevertheless, this concentration was sufficient to significantly reduce vascular structures as observed in the co-culture approaches for both MSC/OEC and MG63/OEC. This observation was associated with a reduction of VEGF and Angiopoietin-2 on the protein level as proangiogenic key molecules inducing the formation of new vascular structures.

In addition, SDF-1 protein concentrations were significantly lowered in response to fucoidan as well as the corresponding receptor CXCR4. In this context, we have recently shown that MSCs are the main producer of SDF-1 [12] in such co-cultures whereas OEC show an expression of the corresponding receptor CXCR4. Thus, fucoidan has the potential to modulate this pathway of cellular crosstalk in normal bone physiology. In the context of osteosarcoma several studies have reported that VEGF and SDF-1 are associated with poor prognosis in osteosarcoma [37,52,53] thus resulting in approaches to counteract this growth factor and chemokine as a therapeutical option for osteosarcoma treatment [54].

Besides VEGF and SDF-1 also elevated Ang-2 levels produced by tumor vessels are considered as a critical factor in the tumor microenvironment. Beyond its direct effect on new blood vessel formation by endothelial cells, Ang-2 also activates proangiogenic properties of circulating mononuclear cells [55,56] which serve as supporting cells in blood vessel formation especially in the context of a tumor environment. Accordingly, Ang-2 is also associated with increased inflammation and metastasis in a variety of tumors [57].

The control of factors such as VEGF, Ang-2 and SDF-1 in the tumor mircroenvironment for instance by fucoidans as natural sulfated polysaccharides should provide a clinical value for osteosarcoma. Nevertheless, the specific action of fucoidan subfractions and the mode of application need further evaluation. According to the literature other groups also reported effects of fucoidan and in particular of fucoidan with low molecular weight on VEGF levels in tumor models [58,59], as well as effects of fucoidan on SDF-1 levels [35]. Nevertheless, contradictory reports on the effect of fucoidan on angiogenesis exist [60]. Using a hydrogel drug delivery approach for medium molecular weight fractions of fucoidan, a recent study reported proangiogenic effects probably associated with locally higher VEGF concentrations and a more continuous release profile mediated by fucoidan. These observations were probably due to the binding capacity of fucoidan for VEGF in the hydrogel [61] thus resulting in a locally higher VEGF concentrations and enhanced vascularization. This study nicely highlights an application dependent action of fucoidan and together with other studies on fucoidan on endothelial progenitor cells [62], these studies might serve as a basis for using fucoidan for proangiogenic therapies related to tissue engineering and regenerative medicine. In contrast, in our study the fucoidan was added to the cultures medium thus lowering the freely accessible VEGF for the cells in culture and finally leading to the reduction of vascular structures. Overall the heparin-like structure [63] and abilities of fucoidan to bind VEGF [64] ensures a wide application of these compounds to modulate angiogenesis but has to be evaluated in a context and application dependent manner. In the present study the free available VEGF and SDF-1 protein levels were reduced resulting in reduction of angiogenesis in response to low dose of fucoidans, whereas the cytotoxic effects were limited.

## Figures and Tables

**Table 1:** Primer list.

Gene Name	Primer Assay Name	Catalogue Number
ALP	Hs_ALPL_1_SG QuantiTect Primer Assay	QT00012957
Osteocalcin	Hs_BGLAP_1_SG QuantiTect Primer Assay	QT00232771
Collagen I	Hs_COL1A1_1_SG QuantiTect Primer Assay	QT00037793
vwf	Hs_VWF_1_SG QuantiTect Primer Assay	QT00051975
VE-Cadherin	Hs_CDH5_1_SG QuantiTect Primer Assay	QT00013244
SDF-1	HS_CXCL12_1_SG QuantiTect Primer Assay	QT00087591
CXCR4	Hs_CXCR4_2_SG QuantiTect Primer Assay	QT02311841
ANGPT-1	Hs_ANGPT1_1_SG QuantiTect Primer Assay	QT00046865
ANGPT-2	Hs_ANGPT2_1_SG QuantiTect Primer Assay	QT00100947
VEGF	Hs_VEGFA_2_SG QuantiTech Primer Assay	QT01036861
RPL13a	Hs_RPL13A_1_SG QuantiTect Primer Assay	QT00089915
Gene name	Sequence	Annealing temperature
CD 31	for 5′- CCGGATCTATGACTCAGGGACCAT-3′ rev 5′-GGATGGCCTCTTTCTTGTCCAG-3′	55 °C

## Supplementary Materials

The following are available online at www.mdpi.com/1660-3397/15/6/186/s1. MSC/OEC co-cultures on day 7: (**a**) visualized by confocal laser scanning microscopy (Red: VE-Cadherin, green: CXCR4, blue: Nuclei. The scale bar represents 150 μm.); (**b**) Quantitative analysis of area and length of pro-angiogenic structures; (**c**) DNA content, paired *t*-test and relative gene expression of angiogenic factors and endothelial markers, 2 way ANOVA; (**d**) ELISA for VEGF, ANGPT-1 and ANGPT-2 in the supernatant normalized to the DNA content. * *p* < 0.05, ** *p* < 0.01, *** *p* < 0.001. *n* = 3, paired *t*-test.

## Abbreviations

The following abbreviations are used in this manuscript:

ALP	alkaline phosphatase
ANGPT-1	angiopoietin-1
ANGPT-2	angiopoietin-2
BSA	bovine serum albumin
CXCR4	C-X-C chemokine receptor type 4 receptor of SDF-1
DMEM	Dulbecco’s Medium Essential Medium
EGM-2	endothelial cell growth medium-2
ELISA	enzyme linked immunosorbent assay
FBS	fetal bovine serum
MSC	mesenchymal stem cell
ODM	osteogenic differentiation medium
OEC	outgrowth endothelial cell
PBS	phosphate buffered saline
Pen/Strep	penicillin/ streptomycin
SDF-1	stromal cell-derived factor-1
VEGF	vascular endothelial growth factor
vwf	von willebrand factor

## References

[1] Rouwkema J., Rivron N.C., van Blitterswijk C.A. (2008). Vascularization in tissue engineering. Trends Biotechnol..

[2] Sivaraj K.K., Adams R.H. (2016). Blood vessel formation and function in bone. Development.

[3] Schipani E., Maes C., Carmeliet G., Semenza G.L. (2009). Regulation of Osteogenesis-Angiogenesis Coupling by HIFs and VEGF. J. Bone Miner. Res..

[4] Brandi M.L., Collin-Osdoby P. (2006). Vascular biology and the skeleton. J. Bone Miner. Res..

[5] Matsumoto T., Kuroda R., Mifune Y., Kawamoto A., Shoji T., Miwa M., Asahara T., Kurosaka M. (2008). Circulating endothelial/skeletal progenitor cells for bone regeneration and healing. Bone.

[6] Melero-Martin J.M., Dudley A.C. (2011). Concise Review: Vascular Stem Cells and Tumor Angiogenesis. Stem Cells.

[7] Versleijen-Jonkers Y.M.H., Vlenterie M., van de Luijtgaarden A.C.M., van der Graaf W.T.A. (2014). Anti-angiogenic therapy, a new player in the field of sarcoma treatment. Crit. Rev. Oncol. Hematol..

[8] Papetti M., Herman I.M. (2002). Mechanisms of normal and tumor-derived angiogenesis. Am. J. Physiol. Cell Physiol..

[9] Dohle E., Fuchs S., Kolbe M., Hofmann A., Schmidt H., Kirkpatrick C.J. (2011). Comparative study assessing effects of sonic hedgehog and VEGF in a human co-culture model for bone vascularisation strategies. Eur. Cells Mater..

[10] Laschke M.W., Schank T.E., Scheuer C., Kleer S., Shadmanov T., Eglin D., Alini M., Menger M.D. (2014). In vitro osteogenic differentiation of adipose-derived mesenchymal stem cell spheroids impairs their in vivo vascularization capacity inside implanted porous polyurethane scaffolds. Acta Biomater..

[11] Shi Y., Kramer G., Schroder A., Kirkpatrick C.J., Seekamp A., Schmidt H., Fuchs S. (2014). Early endothelial progenitor cells as a source of myeloid cells to improve the pre-vascularisation of bone constructs. Eur. Cells Mater..

[12] Shi Y., Wang F., Tiwari S., Yesilbas M., Steubesand N., Weitkamp J.T., Kluter T., Lippross S., Eglin D., Seekamp A. (2016). Role of myeloid early endothelial progenitor cells in bone formation and osteoclast differentiation in tissue construct based on hydroxyapatite poly(ester-urethane) scaffolds. J. Orthop. Res. Off. Publ. Orthop. Res. Soc..

[13] Kirkpatrick C.J., Fuchs S., Unger R.E. (2011). Co-culture systems for vascularization—Learning from nature. Adv. Drug Deliv. Rev..

[14] Kolbe M., Dohle E., Katerla D., Kirkpatrick C.J., Fuchs S. (2010). Enrichment of outgrowth endothelial cells in high and low colony-forming cultures from peripheral blood progenitors. Tissue Eng. Part C Methods.

[15] Kolbe M., Xiang Z., Dohle E., Tonak M., Kirkpatrick C.J., Fuchs S. (2011). Paracrine effects influenced by cell culture medium and consequences on microvessel-like structures in cocultures of mesenchymal stem cells and outgrowth endothelial cells. Tissue Eng. Part A.

[16] Mori N., Nakasone K., Tomimori K., Ishikawa C. (2012). Beneficial effects of fucoidan in patients with chronic hepatitis C virus infection. World J. Gastroenterol..

[17] Prokofjeva M.M., Imbs T.I., Shevchenko N.M., Spirin P.V., Horn S., Fehse B., Zvyagintseva T.N., Prassolov V.S. (2013). Fucoidans as potential inhibitors of HIV-1. Mar. Drugs.

[18] Han Y.S., Lee J.H., Lee S.H. (2015). Antitumor Effects of Fucoidan on Human Colon Cancer Cells via Activation of Akt Signaling. Biomol. Ther..

[19] Hsu H.Y., Lin T.Y., Wu Y.C., Tsao S.M., Hwang P.A., Shih Y.W., Hsu J. (2014). Fucoidan inhibition of lung cancer in vivo and in vitro: Role of the Smurf2-dependent ubiquitin proteasome pathway in TGFbeta receptor degradation. Oncotarget.

[20] Oliveira C., Ferreira A.S., Novoa-Carballal R., Nunes C., Pashkuleva I., Neves N.M., Coimbra M.A., Reis R.L., Martins A., Silva T.H. (2017). The Key Role of Sulfation and Branching on Fucoidan Antitumor Activity. Macromol. Biosci..

[21] Ruperez P., Ahrazem O., Leal J.A. (2002). Potential antioxidant capacity of sulfated polysaccharides from the edible marine brown seaweed Fucus vesiculosus. J. Agric. Food Chem..

[22] Li X.J., Ye Q.F. (2015). Fucoidan reduces inflammatory response in a rat model of hepatic ischemia-reperfusion injury. Can. J. Physiol. Pharmacol..

[23] Silva T.M., Alves L.G., de Queiroz K.C., Santos M.G., Marques C.T., Chavante S.F., Rocha H.A., Leite E.L. (2005). Partial characterization and anticoagulant activity of a heterofucan from the brown seaweed Padina gymnospora. Braz. J. Med. Biol. Res..

[24] Silva T.H., Alves A., Popa E.G., Reys L.L., Gomes M.E., Sousa R.A., Silva S.S., Mano J.F., Reis R.L. (2012). Marine algae sulfated polysaccharides for tissue engineering and drug delivery approaches. Biomatter.

[25] Ale M.T., Mikkelsen J.D., Meyer A.S. (2011). Important determinants for fucoidan bioactivity: A critical review of structure-function relations and extraction methods for fucose-containing sulfated polysaccharides from brown seaweeds. Mar. Drugs.

[26] Fitton J.H., Stringer D.N., Karpiniec S.S. (2015). Therapies from Fucoidan: An Update. Mar. Drugs.

[27] Patankar M.S., Oehninger S., Barnett T., Williams R.L., Clark G.F. (1993). A revised structure for fucoidan may explain some of its biological activities. J. Biol. Chem..

[28] Nishino T., Nishioka C., Ura H., Nagumo T. (1994). Isolation and partial characterization of a novel amino sugar-containing fucan sulfate from commercial Fucus vesiculosus fucoidan. Carbohydr. Res..

[29] Dithmer M., Fuchs S., Shi Y., Schmidt H., Richert E., Roider J., Klettner A. (2014). Fucoidan Reduces Secretion and Expression of Vascular Endothelial Growth Factor in the Retinal Pigment Epithelium and Reduces Angiogenesis In Vitro. PLoS ONE.

[30] Beamer B., Hettrich C., Lane J. (2010). Vascular endothelial growth factor: An essential component of angiogenesis and fracture healing. HSS J..

[31] Hu K., Olsen B.R. (2016). The roles of vascular endothelial growth factor in bone repair and regeneration. Bone.

[32] Kim K.J., Li B., Winer J., Armanini M., Gillett N., Phillips H.S., Ferrara N. (1993). Inhibition of vascular endothelial growth factor-induced angiogenesis suppresses tumour growth in vivo. Nature.

[33] Sankar M.J., Sankar J., Mehta M., Bhat V., Srinivasan R. (2016). Anti-vascular endothelial growth factor (VEGF) drugs for treatment of retinopathy of prematurity. Cochrane Database Syst. Rev..

[34] Schneider T., Ehrig K., Liewert I., Alban S. (2015). Interference with the CXCL12/CXCR4 axis as potential antitumor strategy: Superiority of a sulfated galactofucan from the brown alga Saccharina latissima and fucoidan over heparins. Glycobiology.

[35] Eman R.M., Öner F.C., Kruyt M.C., Dhert W.J.A., Alblas J. (2013). Stromal Cell-Derived Factor-1 Stimulates Cell Recruitment, Vascularization and Osteogenic Differentiation. Tissue Eng. Part A.

[36] Herrmann M., Verrier S., Alini M. (2015). Strategies to Stimulate Mobilization and Homing of Endogenous Stem and Progenitor Cells for Bone Tissue Repair. Front. Bioeng. Biotechnol..

[37] Yu D., Lv F., Zhang J., Li H. (2016). SDF-1 Expression is Associated with Poor Prognosis in Osteosarcoma. Ann. Clin. Lab. Sci..

[38] Qin G., Chen Y., Li H., Xu S., Li Y., Sun J., Rao W.U., Chen C., Du M., He K. (2016). Melittin inhibits tumor angiogenesis modulated by endothelial progenitor cells associated with the SDF-1α/CXCR4 signaling pathway in a UMR-106 osteosarcoma xenograft mouse model. Mol. Med. Rep..

[39] Gil M., Seshadri M., Komorowski M.P., Abrams S.I., Kozbor D. (2013). Targeting CXCL12/CXCR4 signaling with oncolytic virotherapy disrupts tumor vasculature and inhibits breast cancer metastases. Proc. Natl. Acad. Sci. USA.

[40] Katoh H., Hosono K., Ito Y., Suzuki T., Ogawa Y., Kubo H., Kamata H., Mishima T., Tamaki H., Sakagami H. (2010). COX-2 and Prostaglandin EP3/EP4 Signaling Regulate the Tumor Stromal Proangiogenic Microenvironment via CXCL12-CXCR4 Chemokine Systems. Am. J. Pathol..

[41] Liekens S., Schols D., Hatse S. (2010). CXCL12-CXCR4 Axis in Angiogenesis, Metastasis and Stem Cell Mobilization. Curr. Pharm. Des..

[42] Jin J., Zhao W.C., Yuan F. (2013). CXCR7/CXCR4/CXCL12 Axis Regulates the Proliferation, Migration, Survival and Tube Formation of Choroid-Retinal Endothelial Cells. Ophthalmic Res..

[43] Guerin E., Sheridan C., Assheton D., Kent D., Wong D., Grant M., Hiscott P. (2008). SDF1-alpha is associated with VEGFR-2 in human choroidal neovascularisation. Microvasc. Res..

[44] Eklund L., Saharinen P. (2013). Angiopoietin signaling in the vasculature. Exp. Cell Res..

[45] Fuchs S., Hermanns M.I., Kirkpatrick C.J. (2006). Retention of a differentiated endothelial phenotype by outgrowth endothelial cells isolated from human peripheral blood and expanded in long-term cultures. Cell Tissue Res..

[46] Fuchs S., Jiang X., Schmidt H., Dohle E., Ghanaati S., Orth C., Hofmann A., Motta A., Migliaresi C., Kirkpatrick C.J. (2009). Dynamic processes involved in the pre-vascularization of silk fibroin constructs for bone regeneration using outgrowth endothelial cells. Biomaterials.

[47] Moens S., Goveia J., Stapor P.C., Cantelmo A.R., Carmeliet P. (2014). The multifaceted activity of VEGF in angiogenesis—Implications for therapy responses. Cytokine Growth Factor Rev..

[48] Dohle E., Fuchs S., Kolbe M., Hofmann A., Schmidt H., Kirkpatrick C.J. (2010). Sonic hedgehog promotes angiogenesis and osteogenesis in a coculture system consisting of primary osteoblasts and outgrowth endothelial cells. Tissue Eng. Part A.

[49] Ho C.-Y., Sanghani A., Hua J., Coathup M., Kalia P., Blunn G. (2014). Mesenchymal Stem Cells with Increased Stromal Cell-Derived Factor 1 Expression Enhanced Fracture Healing. Tissue Eng. Part A.

[50] Shinohara K., Greenfield S., Pan H., Vasanji A., Kumagai K., Midura R.J., Kiedrowski M., Penn M.S., Muschler G.F. (2011). Stromal cell-derived factor-1 and monocyte chemotactic protein-3 improve recruitment of osteogenic cells into sites of musculoskeletal repair. J. Orthop. Res..

[51] Kimura R., Rokkaku T., Takeda S., Senba M., Mori N. (2013). Cytotoxic effects of fucoidan nanoparticles against osteosarcoma. Mar. Drugs.

[52] Jerez S., Araya H., Thaler R., Charlesworth M.C., López-Solís R., Kalergis A.M., Céspedes P.F., Dudakovic A., Stein G.S., van Wijnen A.J. (2017). Proteomic Analysis of Exosomes and Exosome-Free Conditioned Media From Human Osteosarcoma Cell Lines Reveals Secretion of Proteins Related to Tumor Progression. J. Cell. Biochem..

[53] Benslimane-Ahmim Z., Pereira J., Lokajczyk A., Dizier B., Galy-Fauroux I., Fischer A.-M., Heymann D., Boisson-Vidal C. (2017). Osteoprotegerin regulates cancer cell migration through SDF-1/CXCR4 axis and promotes tumour development by increasing neovascularization. Cancer Lett..

[54] Zhao J., Zhang Z.-R., Zhao N., Ma B.-A., Fan Q.-Y. (2015). VEGF Silencing Inhibits Human Osteosarcoma Angiogenesis and Promotes Cell Apoptosis via PI3K/AKT Signaling Pathway. Cell Biochem. Biophys..

[55] Lewis C.E., de Palma M., Naldini L. (2007). Tie2-Expressing Monocytes and Tumor Angiogenesis: Regulation by Hypoxia and Angiopoietin-2. Cancer Res..

[56] DuBois S., Demetri G. (2007). Markers of angiogenesis and clinical features in patients with sarcoma. Cancer.

[57] Tait C.R., Jones P.F. (2004). Angiopoietins in tumours: the angiogenic switch. J. Pathol..

[58] Chen M.-C., Hsu W.-L., Hwang P.-A., Chou T.-C. (2015). Low Molecular Weight Fucoidan Inhibits Tumor Angiogenesis through Downregulation of HIF-1/VEGF Signaling under Hypoxia. Mar. Drugs.

[59] Huang T.-H., Chiu Y.-H., Chan Y.-L., Chiu Y.-H., Wang H., Huang K.-C., Li T.-L., Hsu K.-H., Wu C.-J. (2015). Prophylactic Administration of Fucoidan Represses Cancer Metastasis by Inhibiting Vascular Endothelial Growth Factor (VEGF) and Matrix Metalloproteinases (MMPs) in Lewis Tumor-Bearing Mice. Mar. Drugs.

[60] Ustyuzhanina N.E., Bilan M.I., Ushakova N.A., Usov A.I., Kiselevskiy M.V., Nifantiev N.E. (2014). Fucoidans: Pro- or antiangiogenic agents?. Glycobiology.

[61] Purnama A., Aid-Launais R., Haddad O., Maire M., Mantovani D., Letourneur D., Hlawaty H., le Visage C. (2015). Fucoidan in a 3D scaffold interacts with vascular endothelial growth factor and promotes neovascularization in mice. Drug Deliv. Transl. Res..

[62] Boisson-Vidal C., Zemani F., Caligiuri G., Galy-Fauroux I., Colliec-Jouault S., Helley D., Fischer A.M. (2007). Neoangiogenesis Induced by Progenitor Endothelial Cells: Effect of Fucoidan from Marine Algae. Cardiovasc. Hematol. Agents Med. Chem..

[63] De Azevedo T.C.G., Bezerra M.E.B., Santos M.d.G.d.L., Souza L.A., Marques C.T., Benevides N.M.B., Leite E.L. (2009). Heparinoids algal and their anticoagulant, hemorrhagic activities and platelet aggregation. Biomed. Pharm..

[64] Lake A.C., Vassy R., di Benedetto M., Lavigne D., le Visage C., Perret G.Y., Letourneur D. (2006). Low Molecular Weight Fucoidan Increases VEGF165-induced Endothelial Cell Migration by Enhancing VEGF165 Binding to VEGFR-2 and NRP1. J. Biol. Chem..

