# Supplementary material for: Crude Fucoidan Extracts Impair Angiogenesis in Models Relevant for Bone Regeneration and Osteosarcoma via Reduction of VEGF and SDF-1
Source: Mar Drugs. 2017 Jun 20;15(6):186. doi: 10.3390/md15060186 (PMC5484136; doi:10.3390/md15060186)
Supplement: Supplementary file 1 [file marinedrugs-15-00186-s001.pdf]

Supplementary Materials: Crude Fucoidan Extracts Impair Angiogenesis in Models Relevant for Bone Regeneration and Osteosarcoma via Reduction of VEGF and SDF-1

Fanlu Wang, Harald Schmidt, Dijana Pavleska, Thees Wermann, Andreas Seekamp and Sabine Fuchs

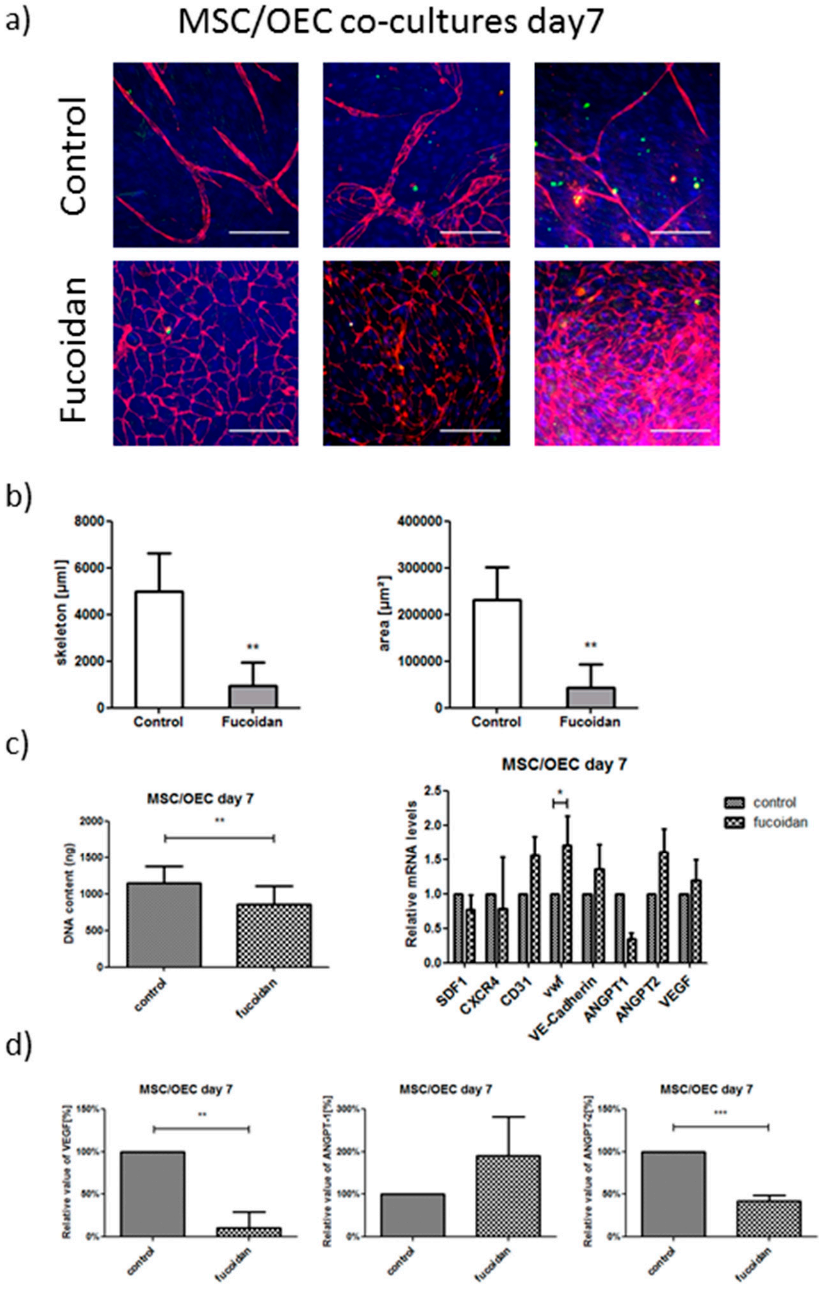

Figure S1. MSC/OEC co-cultures on day 7: (**a**) visualized by confocal laser scanning microscopy (Red: VE-Cadherin, green: CXCR4, blue: Nuclei. The scale bar represents 150 µm.); (**b**) Quantitative analysis of area and length of pro-angiogenic structures; (**c**) DNA content, paired *t*-test and relative gene expression of angiogenic factors and endothelial markers, 2 way ANOVA; (**d**) ELISA for VEGF, ANGPT-1 and ANGPT-2 in the supernatant normalized to the DNA content. * $p < 0.05$, ** $p < 0.01$, *** $p < 0.001$. $n = 3$, paired *t*-test.
